# Influenza and Pneumonia Rampant

**Published:** 1918-11-23

**Authors:** 


### INFLUENZA AND PNEUMONIA RAMPANT.

### Five Days Without a Nurse.

IN a village with upwards of one hundred inhabitants, which has no doctor and is some miles from a railway station, influenza suddenly appeared. All the men of military age have gone to the war, and the village population has taken the war very seriously, for the absence of men has added much to the difficulties of life in this village of Sudborough, Northants. It is interesting to note that all through the war the village church has been open for services from half-past five A.M., the Sacrament is administered on several days in the week, and is attended by many men and women on their way to work at the ironstone furnaces. The Rector, the Rev. Canon Lawson, and his daughter have devoted themselves to the village, its requirements and difficulties throughout the war. When the influenza came it spread rapidly, and the position became more distressing and heart-rending every day. No nurse could be secured, and in desperation a telegram was sent to the Editor of THE HOSPITAL, asking him to send nursing help to save the lives of the younger women and the children who were especially affected.

### ADMISSION OF HOSPITAL VISITORS REFUSED.

This case of war to the death in the Homeland appealed to us, as it would appeal to every reader of THE HOSPITAL, and we set to work with a will to find a woman of intelligence, with a big heart, who had had experience of influenza, that we might send her down and place the village under her care. How Florence Nightingale would have welcomed such an opportunity to show what organisation and nursing skill could accomplish by bringing succour and comfort to the sorely afflicted villagers, the relief of their ailments and, with God's blessing, their restoration to health! There are many Florence Nightingales in the nursing profession in the present day, and at first sight it seemed an easy task for the Editor to find not one but twenty nurses glad of the opportunity to lend a hand in such an emergency as this. In touch as we are with those responsible for the training of nurses and with institutions connected with them, on the first day of our efforts we communicated with most of the chief centres. Everywhere the condition was the same—not a single nurse free for outside work, a great shortage of nurses causing a serious reduction in the staff, owing to the demands of war-work and further outbreaks of influenza. The effect of the latter will be recognised when we state that in one large hospital, with a total nursing staff of 360, 108 were off duty suffering from influenza. The ravages of this disease were serious, and the continuous spread of it led the hospital authorities to take the drastic step of suspending the admission of visitors to the patients. This wise move was fruitful in results, and within fourteen days the spread was arrested, the number of new cases grew fewer and fewer, and at the present time the hospital staff and its inmates are convalescing satisfactorily.

### THE PARAMOUNT CLAIMS OF HOMELAND WORKERS.

Our readers will readily appreciate the strain cast upon the depleted staffs of the hospitals who had the responsibility, worry, and continuous work of discharging the duties of at least one other person as well as their own, which they did here, and are doing, as British nurses always do, without complaint and with zealous devotion worthy of all praise. The present epidemic has affected many hospitals throughout the land, and is one more proof of what we have continuously urged—*i.e.* the paramount claims of the workers who have remained on duty in the Homeland. There the claims upon their services have been paramount indeed, for not only have the needs of the civil population to be met—and the nurses' cases were mainly acute and serious owing to the pressure of war patients on every voluntary hospital—but in these latter months the war cases from the Front have been usually more serious, if possible, than in the early days of the war. We do most earnestly hope that those who have the privilege and responsibility of selecting recipients for honours and medals will bear continuously and specially in mind the claims of these Homeland workers, the inestimable services they have rendered, and the pluck and high sense of duty they have everywhere exhibited.

### THE SECOND DAY A BLANK.

On the second day we continued our search for a nurse for the hard-pressed villagers at Sudborough. Greatly daring we approached the Red Cross. Miss Swift, the Matron-in-Chief of the Joint War Committee, and Miss Easton, Chief of the Home Service Department, were all sympathy and willingness to help our village. But so great had been the pressure that not only was there not available a single one of upwards of 2,000 nurses, but there was not one of the older nurses with whom they keep in constant touch that was free to lend a hand in the cruel emergency we were working to overcome. Indeed, the Red Cross had been so hard pressed that it had to send twenty telegrams that morning, before we telephoned them, to express regret that nurses already promised in various directions could not be supplied as agreed. It was exactly the same at each of the hospitals of high and low degree. Everywhere the demand for nurses was overwhelming, and the font of supply exhausted. So much so that at one great hospital there were urgent calls from members of their medical staff, of the committee, from constant and important supporters of the institution, workers in the cause of the hospital of high and low degree, and other intimate friends, many of them

**Influenza and Pneumonia Rampant**—(*continued*).

serious cases, all of whom had sent within the last few days for nursing aid, none of which could be given. We next tried the Co-operations, and what we may call our own particular hospitals, where the regret was great but to supply a nurse was impossible. One lady who has devoted her life to nursing, the head of a great Co-operation, declared she had never experienced anything like the pressure for nurses in the last three weeks in the whole of her forty years' experience.

### Self-Control and Courage Essential.

Here we may give one caution. The universal experience was that the country everywhere needed the existence, discipline, and help of a Ministry of Health, in touch with the people, inculcating simple rules, and the vital importance of self-control and courage, for the absence of these things had ministered materially to the spread of the epidemic, and, it is not too much to say in some cases, to the severity of the attack. The acuteness of the present epidemic, the rapidity of its spread, and the absence of anything like a sufficient number of trained nurses, is emphasised by the fact that as the urgency increased the rates of payment offered increased too. But all the money in the Bank of England could not bring the richest of the rich the nursing help of which many of them were urgently in need.

### Five Days' Search and One Nurse.

Defeated, but not overcome, we persevered, and had to end our search on the second day with the assurance of a number of institutions that the first available nurse on whom they could put their hands should be reserved for us to send to the relief of the devoted residents in the village of Sudborough. The day concluded with a letter from Sudborough Rectory giving an account of the epidemic, its serious nature, and the number of fatal cases. We have only space to quote one: In the family of one of the principal inhabitants, where the father is a man famous for his industry and hard work, were two daughters. First one was taken seriously ill, and, after a hard fight, thanks largely to the successful and self-denying efforts of the practitioner in attendance, came safely through. Then the second daughter was attacked. Her case in comparison with that of her sister appeared to the mother to be light, and she naturally was less anxious about her. But a peculiarity of many cases of the present epidemic is that the patients grow rapidly worse, and it was so with this poor girl, who fell out of bed and fainted, the window being open. Short-handed as the village was there was no one immediately at hand to realise what had happened. When she was found she was in a most serious state, pneumonia rapidly extended, and the poor girl only lived thirty-six hours. The doctor, who lives some miles away, worked like a horse, but he had other villages and other cases to attend to. All accustomed to the care of the sick will appreciate his devotion and understand how tired and worn the poor man has become. For ourselves there was nothing to be done but to wait another day for reports to come in. On the fourth day we secured a nurse from Darlington, and arranged for her to commence her work on the evening of the fifth day. She was to arrive in the nick of time, for the latest report was that nearly all the villagers have now been attacked, and there only remain three or four more, with the church steeple, for the fiend to ravish. But, alas, late at night came a telegram that she could not be sent, because of her patient's relapse. Then, miraculous to say, a nurse was offered by a near neighbour of ours, and she has gone to Sudborough.

### Another Village Tragedy.

It would appear that very many village communities, large and small, are fighting similar epidemics up and down the country. We have a report from a village in Suffolk, where the type of influenza was unusually severe, and in some cases rapidly fatal, where the inhabitants had the comfort of a resident practitioner. But he, poor man, was attacked by the disease and died. An urgent telegram was sent to St. Thomas's Hospital, where a public-spirited practitioner volunteered to fill the vacancy. He went direct to the village and found his hands full of work. In three days he was stricken. Being a man of resolution and energy he at once took train to London, obtained a bed at St. Thomas's Hospital, and is now, we are glad to say, in a fair way to recover his health. This demonstrates the wisdom and urgency of the course adopted by the Minister of National Service in taking prompt steps to find and increase the number of doctors available for attendance on civil patients throughout the Homeland.

### Some Previous Epidemics.

It may be well to state here, as people are so apt to grow alarmed in the time of a great epidemic, that the influenza outbreaks during the last century occurred at intervals of forty and thirty years. Thirty years ago there was a severe epidemic in London, and a doctor still in practice, then a rising member of the profession, did not know what it was until his father, a physician, told him. The young doctor had it ten times in three years himself, and had two patients at a hotel in Albemarle Street, a recently married couple spending their honeymoon in London. All the servants in the hotel were down with it. There was not a single nurse to be obtained, and this practitioner took his patients in hand as a young man, waited upon them, made their beds, provided them with meals, and brought both of them safely through. He has now as a patient one of their sons, who is home from the war badly wounded. In the West End he had a number of other cases. In one house there were ten people. All the residents but one were smitten. The one who escaped attended to the others with the aid of an old charwoman, who was the only help obtainable. The present epidemic appears to have passed through its severer stages, is now exhibiting a milder form, and seems to be steadily decreasing, ultimately to disappear, no doubt, as it did thirty years ago.

